# Selective local lysis and sampling of live cells for nucleic acid analysis using a microfluidic probe

**DOI:** 10.1038/srep29579

**Published:** 2016-07-14

**Authors:** Aditya Kashyap, Julien Autebert, Emmanuel Delamarche, Govind V. Kaigala

**Affiliations:** 1 IBM Research – Zurich, Säumerstrasse 4, 8803 Rüschlikon, Switzerland

## Abstract

Heterogeneity is inherent to biology, thus it is imperative to realize methods capable of obtaining spatially-resolved genomic and transcriptomic profiles of heterogeneous biological samples. Here, we present a new method for local lysis of live adherent cells for nucleic acid analyses. This method addresses bottlenecks in current approaches, such as dilution of analytes, one-sample-one-test, and incompatibility to adherent cells. We make use of a scanning probe technology - a microfluidic probe - and implement hierarchical hydrodynamic flow confinement (hHFC) to localize multiple biochemicals on a biological substrate in a non-contact, non-destructive manner. hHFC enables rapid recovery of nucleic acids by coupling cell lysis and lysate collection. We locally lysed ~300 cells with chemical systems adapted for DNA or RNA and obtained lysates of ~70 cells/μL for DNA analysis and ~15 cells/μL for mRNA analysis. The lysates were introduced into PCR-based workflows for genomic and transcriptomic analysis. This strategy further enabled selective local lysis of subpopulations in a co-culture of MCF7 and MDA-MB-231 cells, validated by characteristic E-cadherin gene expression in individually extracted cell types. The developed strategy can be applied to study cell-cell, cell-matrix interactions locally, with implications in understanding growth, progression and drug response of a tumor.

Most biological processes are consequences of cells interacting with their microenvironment to perform healthy tissue-level functions^1^,^2^,^3^,^4^. This microenvironment can be the surrounding extracellular matrix or the different cell types constituting the tissue. Cellular interactions predominantly comprise direct physical contact^4^,^5^,^6^, migration^7^ and multimodal signal transduction^8^. Understanding and elaborating the effects of such interactions requires studying cells in their varying microenvironments. Spatially-resolved probing of cells in their native microenvironment in biological substrates, such as complex co-cultures or heterogeneous tissue sections, are therefore fundamental to understanding cell communication, signaling and growth.

Cell-cell interactions are classified as homotypic and heterotypic. Traditionally, homotypic interactions are studied by culturing cells with a stimuli of interest (e.g., matrix type, soluble factors) and performing genomic, transcriptomic and proteomic studies of the entire clonal population. Heterotypic interactions can be studied by means of co-culturing different cell types in physical or biochemical contact using a range of culture methods^9^,^10^. These heterogeneous culture formats are more representative of native tissue biology^11^ than monocultures. There is therefore a need to develop strategies that allow selective sampling and analysis of multiple cells in culture *in situ*. Ideally, such a strategy should be local, non-invasive, aqueous-based, flexible and interactive.

A few technologies^12^,^13^,^14^,^15^,^16^,^17^,^18^ have demonstrated several of these positive traits for local sampling of cells, but do not have all the necessary attributes to perform spatially-resolved probing of cells. These technologies either present the biological substrate to the device^12^,^13^ or present the device to the biological substrate^14^. The former category includes flow cytometric methods and lab-on-a-chip platforms that flow and analyze cells in channels^15^,^16^. Limitations of such biological substrate-to-device approaches include difficulties in handling native biological substrates, complex culture conditions, and long assay times owing to diffusion-based transport of the signaling molecules being investigated. The device-to-substrate category includes devices that selectively scan and process native biological substrates, such as microfabricated hollow cantilevers – FluidFM^17^,^19^, and platforms that use laser-based ablation for laser capture microdissection (LCM) for example^18^,^20^. The Fluid-FM does not confine chemicals on a substrate and can either inject or aspirate a given chemical, decoupling treatment and sampling. The LCM-based sampling method for adherent cells requires culture on specialized substrates and requires specialized training. One other approach that localizes liquid on a substrate is the chemistrode^21^, which confines the processing chemical using a multiphase system, albeit with direct physical contact between the probe and the substrate.

The MFP technology relies on microfabricated heads and platforms for localizing liquids on biological surfaces using hydrodynamic flows and enables handling, analyzing, and interacting with native biological samples^22^,^23^,^24^. Liquid localization is implemented by simultaneous injection and aspiration of a processing liquid; a principle termed hydrodynamic flow confinement (HFC). The locally-confined processing liquid interacts with the biological substrate without the probe physically contacting the substrate, making this approach non-destructive to the surrounding sample. Local confinement of the liquid over the biological substrate increases the number of spatially resolved investigations that can be performed on the same sample. The spatial resolution is dictated by the microchannel dimensions and the microfluidic technique that defines the shape of the confined liquid^25^. Control over spatial resolution enables the MFP to probe substrate properties at varying levels of complexity, for example, probing single cells or hundreds of cells simultaneously for their molecular characteristics. In addition, real-time visual feedback of the substrate processing allows the user to adapt operating conditions during MFP operation, e.g., the probe-to-surface distance, coordinates of the probe on the substrate, and flow rates. This provides the user a high degree of control and flexibility while operating on delicate biological substrates. The MFP and its variants have been used for patterning protein microarrays and complex chemical gradients^22^,^26^, patterning DNA on surfaces^25^, micro-immunohistochemistry of tissue sections^27^, local perfusion of brain slices^28^, single cell pharmacology^29^, probing enzyme activity^30^, and studying cell interactions in monocultures for mRNA analysis^31^.

In this paper, we exploit a particular variant of the MFP, the vertically-oriented MFP^23^, to perform selective spatial sampling of cells from adherent cultures. With the MFP, we implement hHFC^25^, in which we simultaneously confine multiple liquids on a substrate. We locally and rapidly lyse cells using the inner confinement of the hHFC, while shielding the sampled lysate from the surrounding sample using an outer shaping confinement. This approach of selective sampling using the hHFC is strategic for concentrated lysates free of contaminants and a rapid overall workflow for nucleic acid analysis. Specifically, we use the lysates to study DNA and mRNA of targeted cell subpopulations (Fig. 1). A spatially-resolved genome provides the mutational signature of the cells being studied (spontaneous somatic and clonal mutations for example). A spatially-resolved transcriptome represents the immediate response of selected cells to physical or biochemical stimulus. Methods to interrogate the local transcriptome of a cell culture surface after differential exposure to signals, biochemical or physical, would facilitate a deeper understanding interactions between cells and their microenvironment. To the best of our knowledge, this work is the first demonstration of spatially-resolved sampling from live co-cultures for analysis of DNA and mRNA.

## Results

The quality of nucleic acids is affected by the method used to extract them from cells. We identified four imperatives that need to be fulfilled to adapt standard extraction methods for use with the MFP. The method should (i) not denature the nucleic acid, (ii) not adversely affect the yield of the nucleic acid, (iii) be compatible with all steps in the analysis workflow, and (iv) allow selective lysis and sampling of cell sub-populations. To address the above requirements, we investigated interaction of hHFC with cells, chemical systems for local lysis and the downstream analysis of nucleic acids in the context of using the MFP for local lysis.

### Operational parameterization and liquid handling for local lysis

In basic operation of the MFP, a processing liquid is injected through one aperture onto the substrate, and this liquid along with immersion liquid is aspirated via another aperture at a higher flow rate (e.g., *Q*_A_ (*aspiration flow rate*) = 3 × *Q*_I_ (*Injection flow rate*)). Such a flow configuration results in the processing liquid being enveloped by the aspirated immersion liquid. Implementation of the HFC in this configuration for lysing and sampling purposes results in the dilution of the retrieved lysate in the aspirate. This dilution impedes action of the chemistry used for lysing the cells in the aspirate.

**Figure 2:**
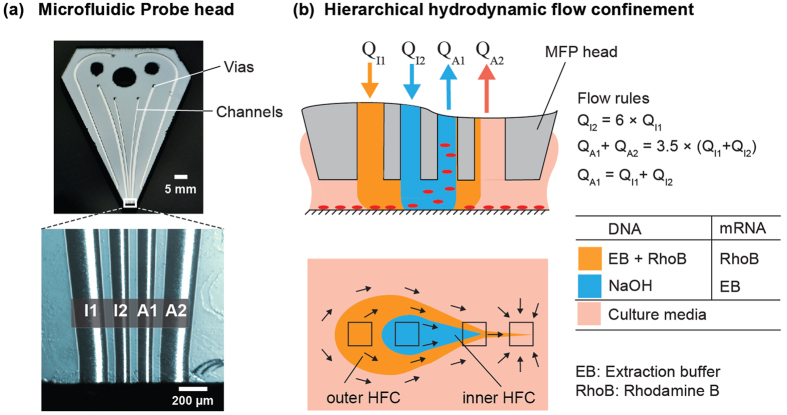
Operational parameters and liquid handling for local lysis of adherent cells using the MFP. (**a**) Photograph of a MFP head. (**b**) Scheme showing the use of hHFC to confine solutions required for cell lysis.

In contrast, the hHFC^25^ (see Fig. 2) uses two injection (I1 and I2) and two aspiration (A1 and A2) apertures to confine two liquids on the substrate. Therefore, the outer HFC nests and confines the processing liquid in the inner HFC. This nesting of liquid relaxes the requirement of a higher aspiration flow rate in the inner aspiration aperture (A1), allowing nearly equal injection and aspiration flow rates (i.e., 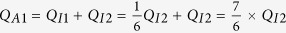), therefore providing an optimal flow strategy for local sampling. In the context of such local sampling, the chemical components of the hHFC have multiple functions. The processing liquid injected from I2 is the component of the hHFC that performs cell lysis, whereas the shaping liquid injected from I1 shields the processing liquid from collecting cell and debris from beyond the hHFC. We note that the continuous flow of the processing liquid, which is inherent to the hHFC, enables simultaneous lysis and sample collection.

Local cell lysis depends on multiple parameters, for example, the time spent by the processing liquid in contact with cells, the chemical system (see next paragraph) and flow velocities (see Fig. S3). These parameters are coupled and here we discuss their contribution in the context of local lysis. We define the time spent by the processing liquid interacting with the adherent cells causing complete cell lysis as the residence time (*T*_R_). *T*_R_ strongly depends on the choice of the lysing chemicals, for example, trypsin often used to detach adherent cells from culture is a weak lysing agent, and would lead to higher *T*_R_. As expected, *T*_R_ also depends on flow velocities - a lower flow velocity results in lower shear on the cell surface, therefore a higher *T*_R_. However, in the context of the lysate concentration, the higher the *T*_R_, the larger is the sample collection volume. This implies that a tradeoff is needed between the flow rates and the lysing chemicals to obtain concentrations of the nucleic acids that can be analyzed. We investigated these coupled parameters using MCF7 cells monolayers as a model system to establish operational parameters.

To obtain DNA that can be used for quantitative analysis using qPCR, we adapted the heat-induced alkaline retrieval method^32^. This method uses sodium hydroxide (NaOH) to simultaneously extract and lyse cultured cells. NaOH facilitates lysis by locally changing the pH on the cells, thus facilitating denaturation of cellular components. However, denaturation of proteins using NaOH leads to a slurry, which can cause perturbations in the hHFC. We therefore choose the flow rates such that the lysate is primed with a small volume of extraction buffer containing Proteinase K (provided by channel I1), resulting in digestion of the slurry in the lysate. The flow rules for obtaining the chemical configuration described are shown in Fig. 2b.

**Figure 3:**
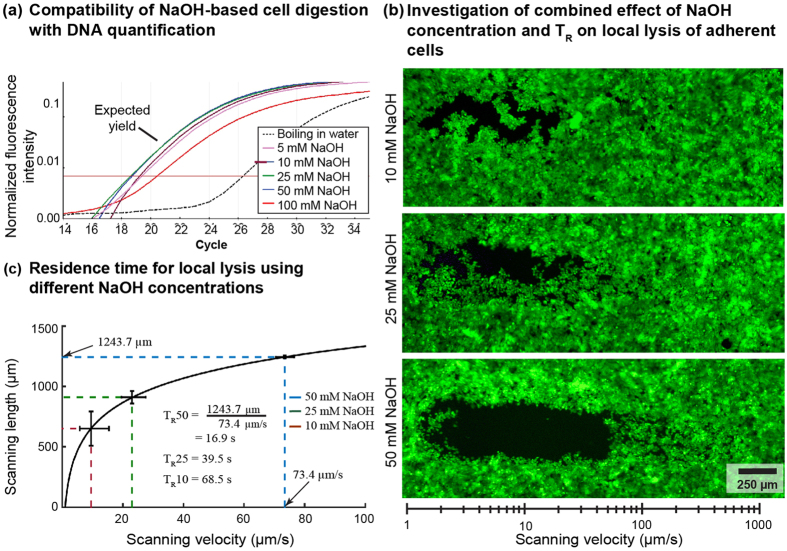
Optimization of chemistry and scan velocity of the MFP for local lysis and digestion of adherent cells. (**a**) Amplification plots of qPCR of a 1000-cell lysate with increasing concentrations of NaOH. (**b**) Lysis lanes using NaOH with an exponential gradient of scanning velocities as shown in the ruler. (**c**) The residence time for different NaOH concentrations were evaluated using (**b**). Outer HFC: Proteinase K and rhodamine B; MCF7 cells cultured to confluence stained with cell tracker green. (n = 3 for qPCR and RT calculation experiments; error bars show standard deviation).

NaOH concentration required for rapid lysis was determined by first establishing the range of concentrations compatible with quantitative PCR (qPCR). A concentration of 50 mM or less of NaOH when used for digesting 1000 cells led to DNA quantities post amplification similar to pure DNA in solution isolated from 1000 cells using standard protocols (Fig. 3a). This suggests that even though higher concentrations (>50 mM) of NaOH might result in more effective lysis, the resulting ion concentrations are incompatible with qPCR.

We chose the flow rates considering tradeoffs between the flow velocity on the cell monolayer (see Fig. S3), the dilution, *T*_R_, and practicality of operating the hHFC for sampling. The flow confinement is minimally perturbed when the aspiration rates in each aspiration aperture are between 3 and 20 μL/min. A NaOH injection flow rate (*Q*_I2_) of 6 μL/min was considered suitable for continuous operation as higher flow rates provided only an incremental improvement in *T*_R_ (see Fig. S3).

We term the region of the adherent cells that interacts with the processing liquid as the footprint of the HFC. The T_R_ needed for lysis of a single footprint can be used to characterize the interaction of different NaOH concentrations with cells. To establish this T_R_, we injected processing liquid with 10, 25 and 50 mM NaOH to lyse MCF7 cells (Fig. 3b). During lysis, we scanned the probe over the monolayer at exponentially increasing velocities from 1 μm/s to 1000 μm/s. The scanning velocity inversely correlates with T_R_. An exponential velocity profile allowed the use of a range of velocities over short distances to establish the T_R_ for complete local lysis. The effective scanning velocity (v_eff_) is the highest scanning velocity at which lysis occurs reproducibly and can be calculated using the length of the lane that is clear of cells.

We obtained suitable local lysis of cells using 50 mM NaOH as the processing liquid, for which T_R_ and v_eff_ were 16.9 s and 70 μm/s, respectively (Fig. 3b,c). Subsequently, we used T_R_ of 20 s to address sample-to-sample variations. For this T_R_, the lysate volume aspirated in channel A1 for a single footprint is 2.3 μL (i.e., V = Q_A2_ × T_R_ = 7 μL/min × 20 s). This 2.3 μL lysate can be used as sample for PCR-based DNA quantification. Collecting the lysate in such small volumes ensures a high concentration of DNA. The use of DNA isolation methods (for example, for column-based purification) would significantly affect the DNA recovery yield. We therefore circumvent the need for DNA isolation methods by performing crude boiling of the high-pH lysate and use of the neutralized lysate as sample for the qPCR. This ensures that low concentration artefacts are avoided during analysis because the concentration of retrieved DNA is orders of magnitude higher than the sensitivity of the PCR method.

### Versatility in sampling volumes and sampling formats

**Figure 4:**
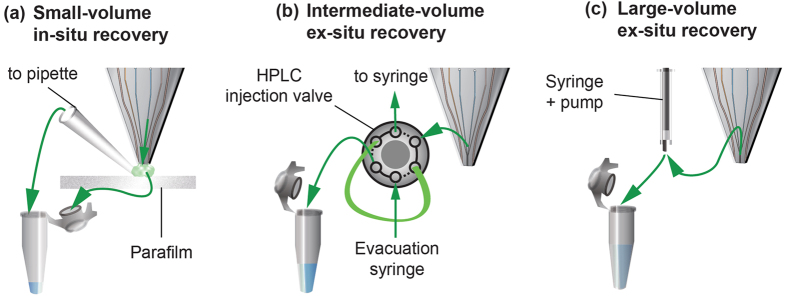
Sample retrieval strategies. (**a**) *In-situ* recovery for small volumes (<25 μL) using immediate purging of liquid. (**b**) *ex-situ* recovery of intermediate volumes (25–100 μL) additionally using an HPLC injection valve, and (**c**) *ex-situ* recovery of large volumes (>100 μL) using syringe collection.

**Figure 5:**
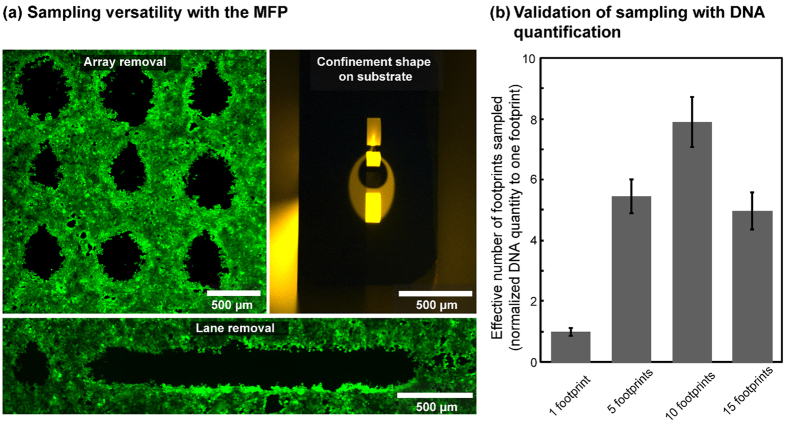
Sampling versatility and quality of extracted DNA using selective cell lysis. (**a**) Fluorescence micrograph of adherent cell layer processed for array and lane cell lysis. (**b**) Quantity of DNA from extracted cells in a fixed volume quantified by qPCR. (n = 5; error bars show standard deviation).

We developed different sample-retrieval strategies to handle a range of sample volumes. For small volumes (<25 μL), we flush the collected lysate onto a Parafilm M® mounted on a solid support or into the cap of a PCR tube (Fig. 4a). For intermediate volumes (25–100 μL), we use a HPLC injection valve and a sampling loop to aspirate through during sampling and bypass while not sampling (Fig. 4b). For large volumes (>100 μL), the sample is directly flushed into a PCR tube (Fig. 4c). With qPCR, we use sample volumes of less than 10 μL; therefore we use the small-volume *in-situ* recovery and collect the lysate in the cap of a PCR tube. We combine local lysis with the scanning capability of the MFP to effect lysis in both array and lane formats with control over the cell quantities sampled (Fig. 5a).

### Quantitation of DNA in local lysate

We sampled multiple footprints (5, 10 and 15), with ~400 cells in each footprint from an MCF7 confluent cell layer and analyzed the DNA quantity in the lysate using qPCR for the *β-actin* gene. A 23 μL lysate (*T*_R_ = 200 s at 7 μL/min aspiration) was collected and quantified from each multiple-footprint sample. The parameter varied in these extractions is the *T*_R_ per footprint and corresponds to 40, 20 and 13 s for 5, 10 and 15 footprints, respectively. Experimental control sample was collected by processing an area on the monolayer already lysed between each multiple-footprint sampling. Controls showed negligible DNA quantities in all experiments.

Each multiple-footprint (5, 10 or 15) sampling was supplemented with a single-footprint extract (*T*_R_ = 200 s) on a given monolayer for normalization. The effective number of footprints for the multiple-footprint extracts was calculated as a multiple of the single-footprint extract, using their respective absolute DNA quantities (mass). DNA quantities increased, as expected, between 1, 5 and 10 footprints, but dropped for 15 footprints (Fig. 5b). The quantity was lower for 15 footprints owing to incomplete lysis of cells within each footprint because of suboptimal *T*_R_.

The quantity of DNA obtained from 10 footprint extracts was comparable to the expected quantity (10 × DNA from 1 footprint), which corroborates *T*_R_ of 20 s. We manually counted cells in 10 footprints prior to local lysis and obtained 369 ± 18 cells in each footprint. qPCR quantification of these 10 footprints led to a DNA equivalent of 307 ± 54 cells/footprint and a 83.2% DNA recovery yield.

The quality and quantity of DNA obtained suggests that (i) the collected lysate contains only the sampled footprints evidenced by the varying quantities of DNA obtained in a fixed volume, (ii) the DNA collected can be amplified and quantified, and (iii) the sampled quantity can be varied based on the requirements of the downstream process.

### Spatially resolved probing of gene expression in adherents co-cultures

Local cell lysis for mRNA extraction requires an adaption of the chemical system compared with the chemical system used for DNA because high pH affects the quality of mRNA. Here we used the extraction buffer containing a combination of detergent (Tween® 20), a chelating agent (EDTA), and a broad spectrum protease (proteinase K). This allows dissolution of the cell membrane, protein digestion, and detachment of the exposed cells, but keeps the nucleic acids intact.

**Figure 6:**
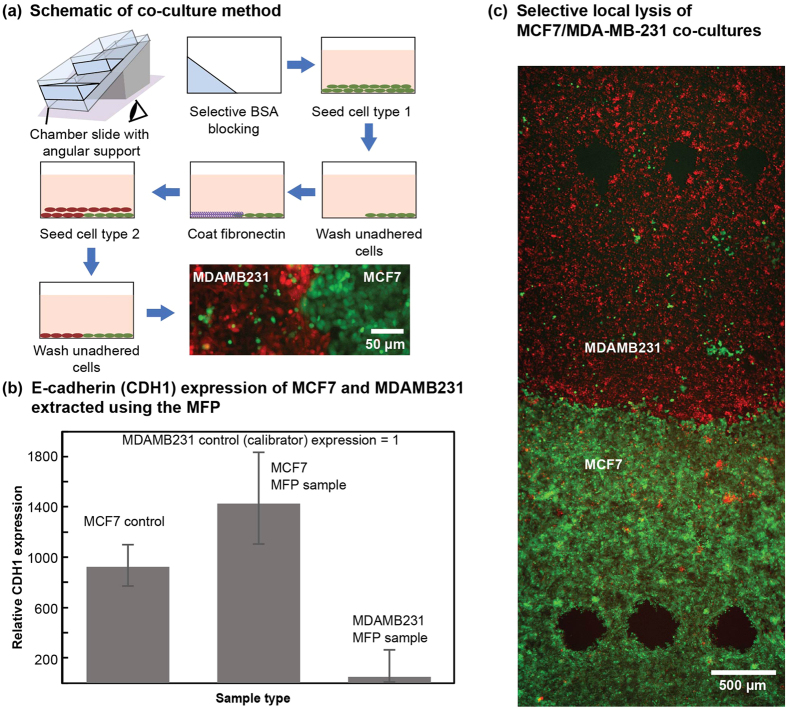
Selective cell lysis and sampling of heterotypic cell cultures for gene expression analysis. (**a**) Scheme of steps to generate a co-culture of MCF7 and MDA-MB-231. (**b**) Representative graph of gene expression in the selectively sampled cells showing higher E-cadherin (CDH1) expression of MCF7 than in MDA-MB-231. (n = 3; error bars show standard deviation), (**c**) Image of the co-cultured substrate post-MFP-based selective sampling of MCF-7 (green) and MDA-MB-231 (red).

To validate local and selective lysis using this chemical system and the quality of the isolated mRNA, we demonstrate lysis of individual cell types in a co-culture of cells with variant expression of a gene. Two phenotypically distinct breast ductal carcinoma cell lines – MCF7 and MDA-MB-231 – were chosen for the co-culture. MCF7 is known to be weakly tumorigenic and migratory *in vivo* with an over-expression of the cell-cell interaction protein E-cadherin (CDH1 gene), whereas MDA-MB-231, a strongly tumorigenic and migratory cell line, has a more mesenchymal phenotype with a marked under-expression of CDH1. We modified a co-culture method developed by Jahaverian *et al*.^33^ to obtain a stable co-culture interface (Fig. 6a) of the two cell types.

Five footprints (~2000 cells) were locally lysed and sampled from each cell type on the substrate (*N* = 2) in 150 μL (*T*_R_ ~4 min). Experimental controls were extracted between each five-footprint sampling by residing on an already sampled spot on the co-culture substrate. RNA was isolated using column-based extraction for each selective lysate, and the sample was introduced as template for rtPCR for CDH1 with β-actin as the normalizing housekeeping gene (n = 3). mRNA was also isolated from monoculture to provide the required controls and calibrators. We were able to obtain a lysate of about 13 cells/μL, prior to RNA reconcentration and isolation using column-based purification.

We observed a 1000-, 1400- and 50-fold over-expression of CDH1 in MCF7 in mono culture, MFP extracts of MCF7 and MDA-MB-231 respectively, normalized with the expression in MDA-MB-231 monoculture (calibrator) (Fig. 6b). The controls showed negligible amplification of the gene. The expression profiles obtained for different cell types validates the mRNA quality and the presence of individual cell types in the lysate.

## Discussion

Selectively probing the genomes and transcriptomes of cells in native biological samples provides a deeper understanding of signaling processes through which cells interact with their microenvironment and transform to diseased states. Molecular biology assays for performing such local multimodal genomic and transcriptomic analyses usually require high concentrations of nucleic acids for artefact-free analysis. This is challenging for local sampling strategies as they inherently contain only a small quantity of nucleic acids. The MFP and the hHFC in conjunction with the chemical system described in this work address this challenge, while providing significant improvements in processing times and local analysis of nucleic acids. The HFC overcomes diffusion limitations that are characteristic of most molecular biology assays performed on bench-top platforms by means of convectively replenishing the liquid processing the surface. In addition, through minimal dilution of the processing liquid, the hHFC enables continuous lysing action on the cells on the substrate and in the aspiration channel, which results in highly concentrated lysates.

The choice of processing liquid has been adapted for each of the nucleic acids to optimize the lysis for downstream analysis. We use NaOH when DNA is the analyte of interest and Proteinase-K-based extraction buffer when mRNA is the analyte of interest. The residence time is the parameter that characterizes the extraction method, as it depends on the chemistry and the flow rates and determines lysate concentration and volume as a consequence. Potential approaches to modulate and tune *T*_R_ based on the required application include recirculation of the chemical system between the inner apertures^34^ during sampling operation, use of alternate chemistries for shorter *T*_R_, modification of the channel geometries, and increase in temperature of operation. *T*_R_ is independent of the size of the area of interest on the cell surface. This attribute to modulate *T*_R_ enables rapid sampling of adherent cells at varying levels of spatial resolution.

Generally, molecular analysis of adherent cells is performed on several different scales, from 3D spheroidal cultures containing hundreds of cells to a single cell, depending on the objective of the investigation. Here, we sample ~400 cells to avoid method-based and statistical artefacts in genomic and transcriptomic studies. With this level of spatial resolution, we were able to obtain quantities of DNA that are sufficient for DNA-sequencing studies^35^,^36^. With respect to mRNA analysis of selectively lysed cells in co-culture, we were able to obtain expected gene expression profiles for the isolated cell types. Because rtPCR was used for the analysis, the quality of the mRNA is compatible with RNAseq studies with appropriate scaling of the spatial resolution. Purified DNA and mRNA can be used for non-PCR based analytical methods such as MALDI-TOF, for improving the molecular resolution of the obtained profiles. The chemical and fluidic configuration presented in this paper may be suitable for probing gene-expression profiles in 3D cultures, as evidenced by the effective local lysis of multilayered cells that were obtained in some cases by over-culture. For the particular case of studying a single cell in its microenvironment, we might be able to scale down the spatial resolution. For example, in our earlier work^23^, we fabricated apertures as small as 1 μm × 1 μm, however with such aperture dimensions we cannot exclude rapid clogging of channels during regular operation. Another approach includes modulating the shaping liquid to have a small fraction of the processing liquid make contact with a surface, as detailed by Autebert *et al*.^25^.

In addition to the probing of local genomes and transcriptomes of cells in culture at the end-point of a given study, we can perform temporal studies as MFP is non-destructive to the un-sampled regions on the cell layer. By allowing chronological investigations on effects of various stimuli, this provides an additional dimension to the information obtained from adherent cell experiments. Furthermore, most molecular biology protocols perform multi-step chemistry on biological substrates. With the hHFC, we can perform such multi-step chemistry by injecting liquids sequentially and furthermore process a surface with different chemicals simultaneously. The laminar flow profiles of the liquids injected on the substrate ensure distinct aspiration of the injected liquids. We developed and chose chemical systems to be compatible with such distinct, but simultaneous sampling. We used the components of the shielding liquid (extraction buffer with proteinase K) in the local DNA analysis experiments as the processing liquid in the local mRNA analysis. Therefore, the developed strategy has the advantage of sampling the inner and the outer HFCs for DNA and RNA analysis simultaneously.

The chemistry used for local lysis causes disintegration of the exposed cell by solubilizing the membrane and the intracellular machinery. Therefore, the strategy developed can be broadly used to study DNA and RNA, not only in fixed population of cells, but also applied to clinical samples so as to obtain spatially-resolved mutation profiles of heterogeneous tissues for diagnostic applications.

### Concluding remarks

We demonstrated a new strategy for spatially-resolved and selective lysis of cells from adherent mono- and co-culture substrates for local DNA and mRNA analysis. This method can further be applied to perform temporally and spatially resolved probing of gene expression in adherent cultures, thus improving our understanding of multicellular interactions significantly. The quality and quantity of the nucleic acids obtained using this strategy enable multiple downstream analyses, exemplified by qPCR and rtPCR in this paper, and eventually next-generation sequencing (DNAseq and RNAseq). Combining next-generation sequencing with the local sampling strategy described here can provide a complete molecular profile of cells interacting with their native microenvironment, with these profiles resolved potentially even to the level of a single molecule.

## Materials and Methods

### MFP platform, head and handling

The MFP platform included motorized scanning stages, peripherals for liquid handling and a microfabricated head. This platform was placed on top of an inverted microscope for real time observation of local lysis and sampling.

The microfabrication of the silicon-glass head and the associated platform have been described elsewhere^23^. Briefly, the head and its channels were designed using L-edit (Tanner EDA), and the channel pattern was written on a chromium mask using a laser writer (WL 2000, Heidelberg Instruments Mikrotechnik GmbH). A 400-μm-thick Si layer was etched to 100 μm depth with the masked pattern using deep reactive ion etching (DRIE) and then anodically bonded to a 500-μm-thick glass wafer, resulting in micron-sized, 100-μm-deep channels. The channels were filled with wax through the vias, and the patterned silicon/glass wafers were diced to form individual heads. The heads were polished, dewaxed, and cleaned prior to use. The heads used for the experiments in this paper had channels of the dimension 200 × 100 μm^2^ (forming the outer apertures), 100 × 100 μm^2^ (forming the inner apertures), and 500 × 100 μm^2^ (forming the immersion channels) with 100-μm spacing between apertures (see Fig. 2).

During operation, the vias in the head were connected to liquid-handling peripherals, which included pumps (Nemesys, Cetoni GmbH, Korbussen, DE), syringes (Hamilton 1705 TLLX, Bonaduz, CH), and associated tubing and connectors (Upchurch Scientific, IDEX Health & Science LLC, Oak Harbor, WA, USA) providing the required fluid flow. The platform was mounted atop the stage of an inverted microscope (Nikon Eclipse TE300, Egg, CH), equipped with a DS Fi2 CCD camera (Nikon, Egg, CH). The high-precision motorized stage (LANG GmbH, Hüttenberg, DE) was connected to the head holder, thereby allowed precise movement in the three axes.

### Cell culture

Human breast-cancer cell lines MCF7 (HTB-22) and MDAMB-231 (HTB-26) were purchased from ATCC and cultured in Dulbecco’s minimal essential medium (DMEM) supplemented with 10% fetal bovine serum, and 1% Pen-strep antibiotic (complete DMEM with supplements). The cells were expanded in T75 flasks at a seeding density of 0.15 M cells/ml and cultured over two days. The cells were harvested after the culture had reached a confluency of 80–90% and seeded at appropriate densities for monoculture or co-culture on chamber slides. All cell-culture consumables were purchased from ThermoFisher Scientific, MA, USA.

MCF7 cells at 0.35 M cells/mL were seeded onto two chamber slides (CS2) (Fisher Scientific, Reinach, CH) and cultured over two days prior to the monoculture experiments. The live/dead viability/cytotoxicity kit for mammalian cells (ThermoFisher Scientific, MA, USA) was used to visualize the lysis of viable cells. The concentrations were in line with the manufacturer’s instructions. 10 μM of Cell tracker green (CMFDA) in DMEM was also used to visualize local lysis.

For co-culture experiments, the protocol described by Javaherian *et al*.^33^ was modified (Fig. 6a). The CS2 were first selectively blocked for half the culture area with 0.5% (w/v) BSA by incubating them over an angular support for 2 h at 37 °C in a humidified incubator. MCF7 cells were seeded at 2 M cells/ml (in 500 μL of DMEM) on the selectively-blocked CS2s for 100 min. The cultured surface was then gently washed, resulting in delamination of the unbound cells on the blocked half of the CS2s. The blocked region was then reactivated by incubating with 50 μg/ml Fibronectin from bovine plasma (Sigma-Aldrich Chemie GmbH, Buchs, CH) for 30 min. Post reactivation, the MCF7s on the CS2 were cultured overnight with complete DMEM. MDAMB-231 in T-flasks and the MCF7 contained in the CS2 were then labeled using 10 μM CMRA and CMFDA dyes in DMEM, respectively, for 45 min. The CMRA-labeled MDAMB-231were then harvested from the T-flasks and seeded at 2M cells/ml (in 500 μL complete DMEM) into the CS2 containing CMFDA-labeled MCF7s and incubated for 180 min. The CS2s were then washed to remove unbound cells and cultured for two days.

### Local lysis, liquid handling and sample retrieval

All experiments performed used the hHFC^25^ with flow rates of 1, 6, −7 and −17.5 μL/min used for I1, I2, A1 and A2 channels, respectively. The flow rates were chosen in line with the flow rules described in Fig. 2.

Varying concentrations of NaOH were used as processing liquid for the characterization and optimization of local lysis with the MFP (Fig. 2). The shielding solution used was 10 μM rhodamine B, 0.5% tween 20, 1 mM EDTA and 10% Proteinase K in 50 mM Tris at pH 8 (Extraction buffer – EB). MCF7 monocultures in CS2 at room temperature were used as the substrate for the studies. Zero leveling was done as described in the literature^23^ on a glass slide, and the MFP was scanned 50 μm above the substrate. The scanning was performed by programing the stages to follow an exponential velocity profile in the *x*-direction.

For the DNA analysis studies, 50 mM NaOH was used as processing liquid and EB as the visualization/shielding liquid. Multiple footprints were sampled in 23 μL, and the lysate was collected by purging it into the cap of a 200 μL PCR tube through A1 (Fig. 2b – small-volume *in-situ* recovery).

For the local RNA analysis studies, EB without rhodamine B was used as the processing liquid and a 10-μM solution of rhodamine B as the shielding/visualization solution. Collected lysate (≈120 μL) was purged directly into a 200 μL PCR tube (Fig. 2b – large-volume *in-situ* recovery). The RNA was then isolated from the lysate using the RNeasy Plus Micro Kit (Qiagen, Hilden, DE) using the manufacturer’s protocol in 12 μL RNase-Free Water.

### DNA and RNA quantification

For quantification of the number of cells within each footprint, we manually counted cell tracker stained cells in ten 100 μm × 100 μm areas on 3 chamber slides. However, a nuclear stain and automated counting is imperative if less than 50 cells are to be sampled. DNA quantification from local lysates was performed using quantitative PCR (qPCR). The NaOH lysates were first boiled for 10 min at 95 °C and then neutralized using 1:1 50 mM tris-Cl at pH 8. The lysate was directly introduced as the template for qPCR. This method leads to high yields of DNA by circumventing the use of column-based isolation. qPCR was performed on the Rotorgene Q thermocycler platform in combination with the Rotor gene SYBR green kit (Qiagen, Hilden, DE) for genomic β-actin using forward primer TCCCTGGAGAAGAGCTACGA and reverse primer AGCACTGTGTTGGCGTACAG, leading to a 194 base pair product.

Cycling conditions were an initial activation step (95 ^o^C for 5 min) followed by 35 cycles of denaturation (95 ^o^C for 5 s) and a combined annealing/extension (60 ^o^C for 10 s). Reaction contents included 50% of 2 × master mix, 1 μM primers, and 4 μL of the lysate in a 20 μL reaction volume. Standard curves for the DNA were obtained for a serial dilution of 10 pg to 10 ng of DNA isolated from cultured MCF7. All samples were run in triplicate.

Extracted lysates were normalized for relative quantification by using a single MFP footprint extraction lysate with every multiple-footprint extraction lysate (5, 10 and 15 footprints in different experiments). The relative quantities of DNA were evaluated by dividing the absolute quantity of the multiple-footprint lysate by the single-footprint lysate. All MFP extractions were run in triplicate, and errors obtained were standard deviation for *n* = 3 for the various footprints.

The yield (obtained/theoretical × 100) for 10-footprint lysates and DNA quantification was calculated by first evaluating the cell number obtained in 10 footprints using a qPCR standard curve for MCF7 cells. The qPCR was performed in triplicate for three 10-footprint extracts obtained of the same experimental cell surface. The theoretical number of cells was obtained by evaluating the area of five lysed footprints and finding the equivalent number of cells using five independent 100 × 100 μm^2^ regions of interest (ROIs) of undisturbed MCF7s cultured on CS2s.

While probing the range of NaOH concentrations compatible with qPCR, DNA from a 1000 cells was extracted using the DNeasy Blood and Tissue Kit (Qiagen, Hilden, DE). All other qPCR parameters were the same as described above.

Relative quantification of E-cadherin expression (CDH1) on the isolated RNA from selective lysates of MCF7 and MDAMB231 MFP was performed by rtPCR on the Rotorgene Q platform in combination with Rotorgene Probe PCR mix (Qiagen, Hilden, DE). PrimeTime mixes from Integrated DNA Technologies (IDT, Iowa, USA) for probe-based rtPCR of CDH1 and ActB were purchased and resuspended to a 20 × stock, which contains ready mixes of probes, forward and reverse primers. The sequences are available on the manufacturer’s website.

Cycling conditions were a reverse transcription step (50 ^o^C for 10 min) followed by the standard two-step cycling PCR as describe above. Reaction contents included 50% 2 × Rotor-Gene Probe rtPCR Master Mix, 5% PrimeTime stock mix (CDH1 or ActB), 1% Rotorgene RT mix, and 2 μL RNA sample in a 20 μL reaction.

The relative quantification was performed by the 2ΔΔCt method^37^ using the RotorGene 2 software provided with the machine, with β-actin (ActB) expression as the normalizing housekeeping gene and expression of MDAMB231 cultured in T-flasks as the calibrator.

## Additional Information

**How to cite this article**: Kashyap, A. *et al*. Selective local lysis and sampling of live cells for nucleic acid analysis using a microfluidic probe. *Sci. Rep.* **6**, 29579; doi: 10.1038/srep29579 (2016).

## Supplementary Material

Supplementary Information

Supplementary Dataset 1

Supplementary Dataset 2

Supplementary Information

## Figures and Tables

**Figure 1:**
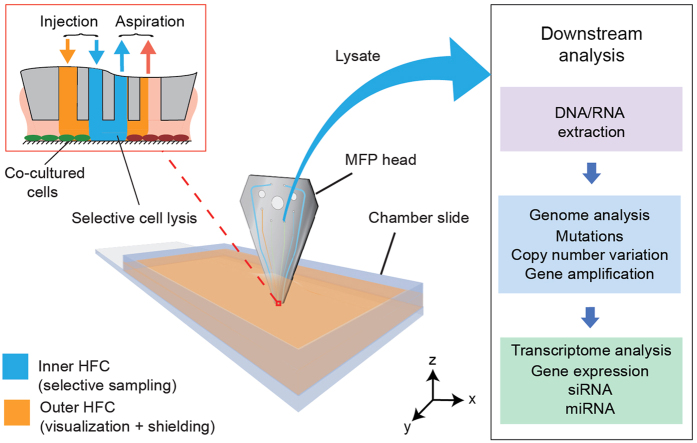
Selective lysis and sampling of cells from monolayers for genomic and transcriptomic profiling. Illustration of a microfluidic probe head scanning over adherent cells on a chamber slide, the interaction of biochemicals on the cells monolayers (inset). The sampled lysate is introduced into workflows for downstream analysis of nucleic acids.
