# Supplementary material for: Selective local lysis and sampling of live cells for nucleic acid analysis using a microfluidic probe
Source: Sci Rep. 2016 Jul 14;6:29579. doi: 10.1038/srep29579 (PMC4944176; doi:10.1038/srep29579)
Supplement: Supplementary Information [file srep29579-s1.pdf]

Selective local lysis and sampling of live cells for nucleic acid analysis using a microfluidic probe

Aditya Kashyap, Julien Autebert, Emmanuel Delamarche, Govind Kaigala*

IBM Research-Zurich, Säumerstrasse 4, CH-8803 Rüschlikon, Switzerland

Supplementary information

Supplementary table: Primer list

S.No	Target	Sequence	Description
1	Cytoplasmic actin isoform X1 (Genome specific)	**F:** GGATGCAGAAGGAGATCACT	Self designed primers for qPCR
		**R:** CGATCCACACGGAGTACTTG	
2	Actin B (ActB): Exon 3-4	**P:** /56-FAM/CCATGTACG/ZEN/TTGCTATCCAGGCTGT/3IABkFQ/	PrimeTime Assay from IDT technologies for rtPCR
		**F:** GCGAGAAGATGACCCAGA	
		**R:** CCAGTGGTACGGCCAGA	
3	E-cadherin (CDH1): Exon 12-13	**P:** /56-FAM/ACCCACCTC/ZEN/TAAGGCCATCTTTGG/3IABkFQ/	PrimeTime Assay from IDT technologies for rtPCR
		**F:** CCATTCAGTACAACGACCCA	
		**R:** CTGGTTATCCATGAGCTTGAGA	

F: Forward primer; R: Reverse primer; P: Probe

Supplementary figure 1: MFP platform

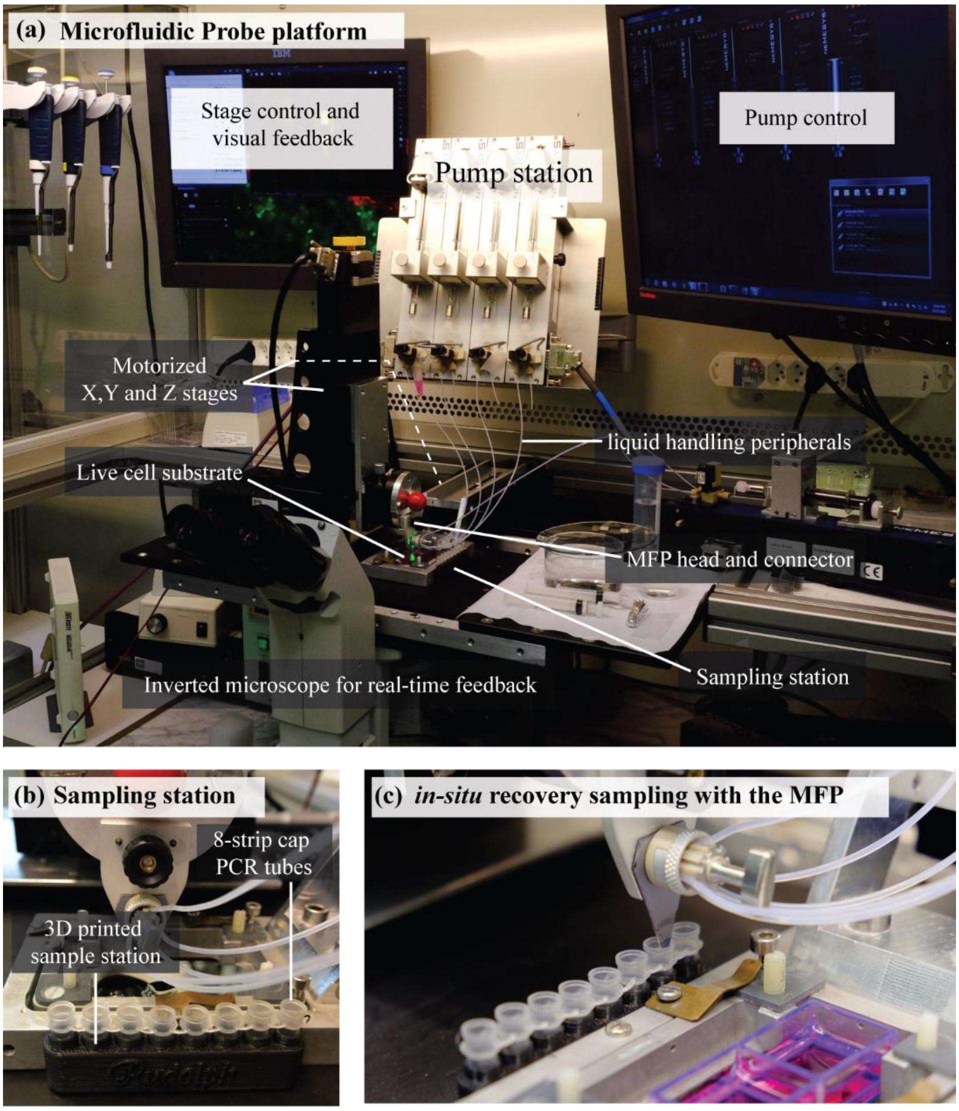

(a) The MFP platform. MFP platform comprises of high precision motorized XYZ scanning stage, peripherals for handling liquids- pumps, syringes and associated tubings, and a microfabricated head. This platform is placed on top of an inverted microscope for real time observation of the local lysis. **(b) Sampling station.** A 3D-printed sampling station is magnetically mounted to the substrate holder, allowing the X, Y stages to position the station under the MFP head for sample recovery. **(c) *in situ* sample recovery.** Recovery of the lysate by purging contents of the channel A1 (see fig 2) into 8 strip PCR tubes, which are then used for qPCR analysis after appropriate preprocessing.

Supplementary figure 2: MFP head design for decellularization

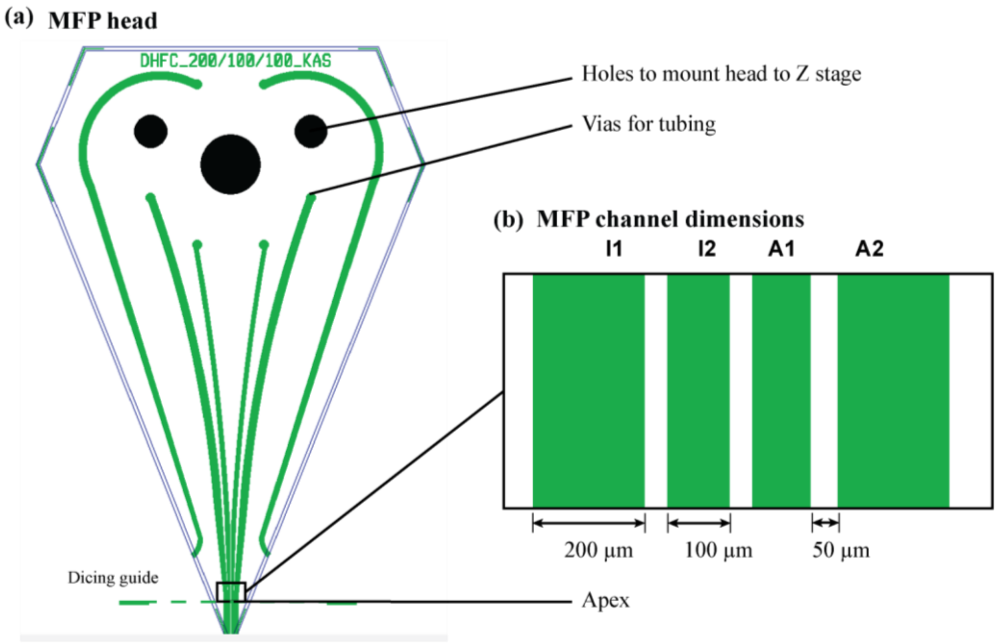

(a) MFP head design. The MFP head designed for decellularization experiments has 6 channels- 2 for injection, 2 for aspiration and 2 for injection of immersion fluid. The different components of the head which interface with the other components in the platform are labeled. The mounting holes are used to connect the MFP head to the Z stage, which allows the control of the gap distance between the substrate and the MFP head. X and Y stages are used to control the substrate holder. (b) Channel dimensions of the MFP head.

Supplementary figure 3: Effect of flow rate on decellularization of cell monolayer

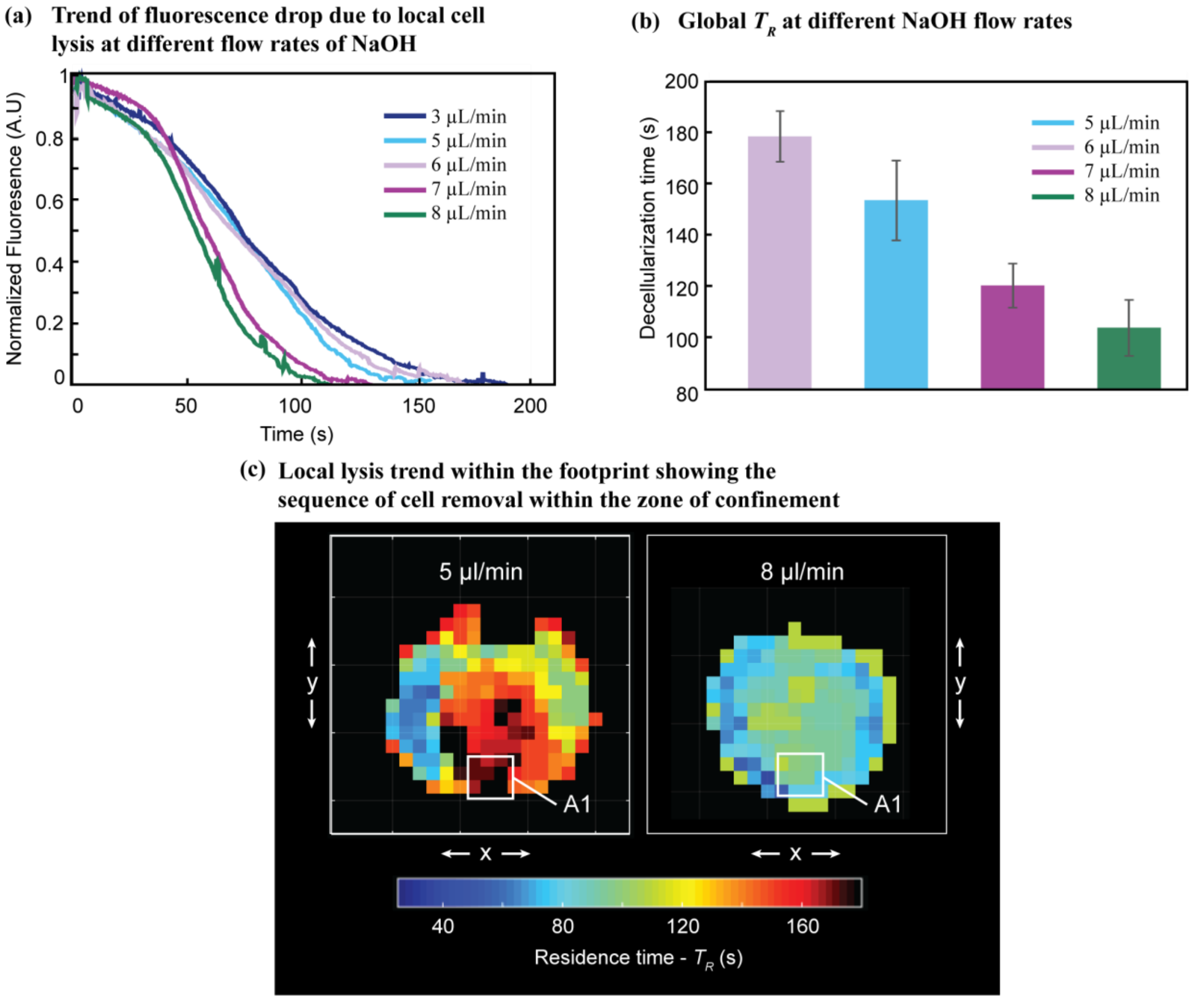

(a) Representative trend of local cell lysis within the footprint for different flow rates evaluated by studying drop in fluorescence of the lysed and sampled cells. **(b)** Time for local lysis evaluated for different NaOH flow rates by analysis of the trends shown in (a). Higher flow rates only provide an incremental improvement in T_R. This observation combined with the observed trends, times and practical considerations explained in the results section allowed us to establish an operational flow rate Q_{i2} of 6 µL/min. **(c)** Analysis of videos that show the temporal sequence of cell removal within the footprint for two different flow rates (imageJ and Matlab analysis). The analysis shows faster removal at the edges of the confinement for both flow rates, which may be a consequence of the gap distance changing as the cells are removed from the substrate which consequently may affect the shear profile. Further studies have to be carried out to understand the underlying mechanism. The T_R across the footprint is more homogeneous for higher flow rates making the sampling procedure more surgical. This observation provides a preliminary insight on the mechanism of action of the processing liquid and the coupling of the flows with the chemistry.

Supplementary video: Local Cell Lysis using the hHFC and the MFP.

Real-time video of local lysis at 10× magnification (2× video speed with 4× magnification in inset). The hHFC with NaOH in the inner confinement lyses and samples cells as the MFP scans over the cell culture substrate. The aperture within which the lysate is sampled, is annotated in the video. Post lysis, the sample is directly purged from the head into a PCR tube.
